# A Case of Meige Syndrome Treated With Baclofen

**DOI:** 10.7759/cureus.10570

**Published:** 2020-09-21

**Authors:** Kimberly Nguyen, Carl Hoegerl

**Affiliations:** 1 Neurology, Liberty University College of Osteopathic Medicine, Lynchburg, USA; 2 Internal Medicine and Neurology, Centra Health and Liberty University College of Osteopathic Medicine, Lynchburg, USA

**Keywords:** meige syndrome, baclofen, blepharospasm, case report

## Abstract

Meige syndrome, also known as blepharospasm-oromandibular dystonia, is a neurological movement disorder that involves the involuntary muscle contractions of the eyes, mouth, tongue, and jaw. It is often associated with other disorders, such as Parkinson’s disease. We describe a case of an 87-year-old man with Meige syndrome who was successfully treated with oral baclofen.

## Introduction

In 1910, Henry Meige, a French neurologist, was the first to describe abnormal facial contractions along the midline of the face and named it “spasm facial median.” Named after him, Meige syndrome is a rare neurological disorder that results in involuntary muscle spasms involving the muscles around the eyes (blepharospasm dystonia) and involuntary muscle contractions of the mouth, tongue, and jaw (oromandibular dystonia) [1].

Blepharospasm is characterized by abnormal and frequent prolonged contractions of the eyelids leading to the inability to open the eyes. Several stimuli that may contribute to the eye irritation include bright lights, environmental factors, and fatigue. Whereas oromandibular dystonia is characterized by forced contractions of the jaw and tongue that result in the inability to eat and to open or close the mouth, other manifestations may include clenching or grinding of the teeth, displacement of the jaw, chin thrusting, or pursing of the lips [2].

The etiology and associations of Meige syndrome remain unknown but have been associated with other disorders, such as tardive dyskinesia, Wilson's disease, and Parkinson's disease [3]. Individuals with Meige syndrome usually fall within the age group of 30-70 years, with a median age of 55.7 years. Females are often more affected than males [4].

The possible pathogenesis of Meige syndrome is hypothesized to be due to the increase in cholinergic and dopaminergic hyperactivity or the decrease in function of inhibitory neurons, like GABAergic neurons. Some individuals who have taken neuroleptic medications for more than a year had altered receptor function that caused cervical and facial dystonia due to heightening central dopaminergic activity and improved with dopaminergic depleting agents [4].

The most effective treatment for Meige syndrome to date is the use of botox injections; however, the effects are not long lasting. However, most patients are often treated with oral medications, such as anticholinergics, tetrabenazine (monoamine storage inhibitors), benzodiazepines, and baclofen [4].

## Case presentation

An 84-year-old man presented to our neurology clinic in December 2018 with a history of episodes of jerking of his face, neck, and mouth that affected his speech and eating. He noticed his symptoms getting worse when trying to eat. Prior to presenting to the clinic, his primary care physician had started chlorpromazine because they suspected a diagnosis of esophageal spasm. He did better for a while but then his symptoms got worse. He would have shaking spells intermittently throughout the day.

Myoclonus was suspected initially but he was not having symptoms at the time of presentation, so this was based on history alone. Every time he came into the clinic, his symptoms were fine. Finally, in January 2019, he presented to the clinic with symptoms that appeared to have gotten worse. Episodes of jerking were more frequent and he had increased difficulties in speech. His symptoms appeared to suggest muscle spasms and tremor rather than myoclonus. He would have difficulties with dysarthria and with dysphagia.

Aside from these signs and symptoms, he denied chills, fever, chest pain, palpitations, cough, shortness of breath, muscle pain, and muscle weakness. His family history revealed that his mother had dementia and hypertension. His father had heart disease and nephrolithiasis.

**Figure 1:**
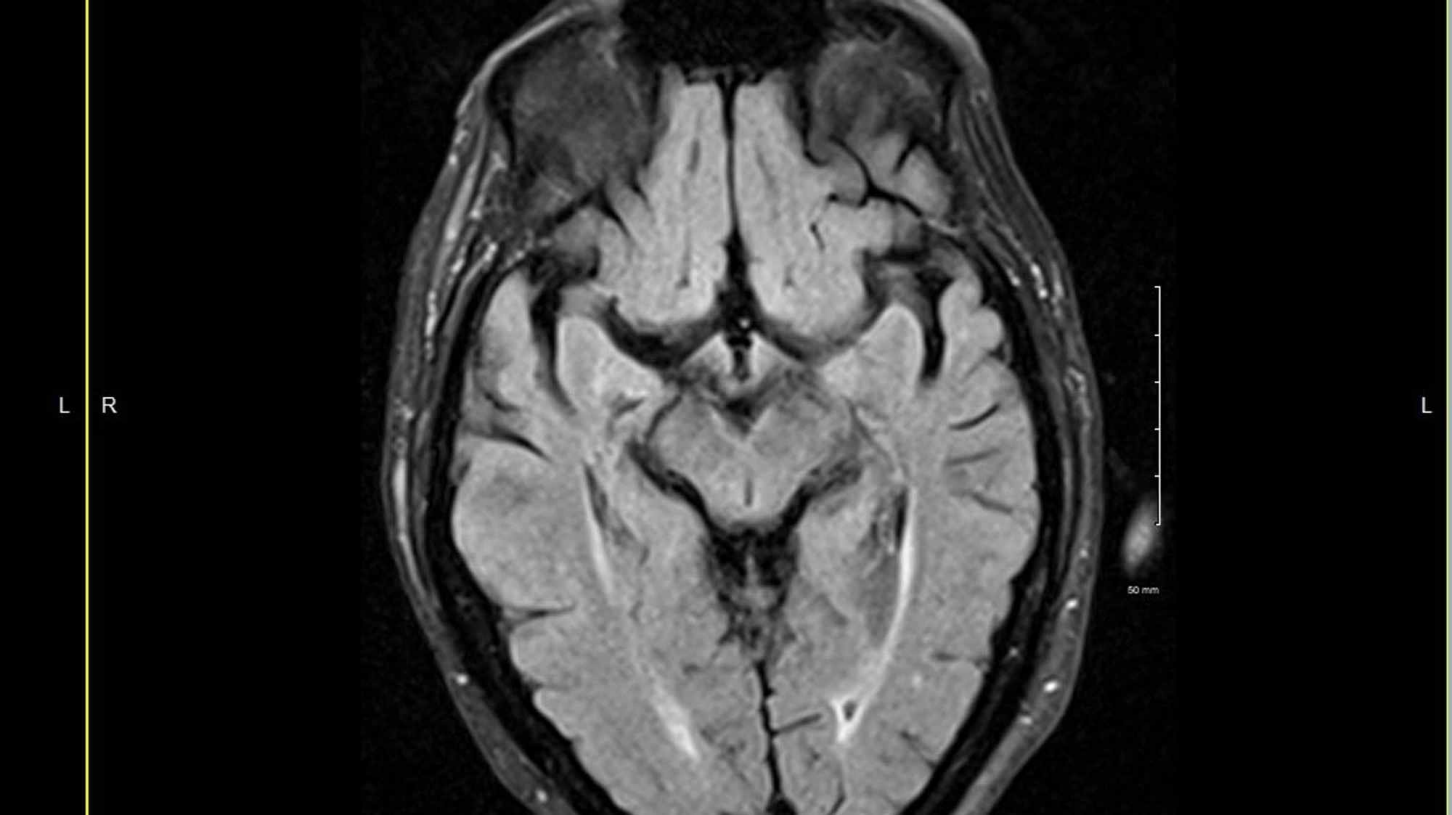
MRI of brain. MRI showing minimal small vessel disease that is not unexpected for age. Otherwise, it was an unremarkable MRI (normal). Electroencephalogram (EEG) was normal as well. No EEG tracings available for publication.

Electroencephalogram (EEG) performed, and this was normal as there was a concern for myoclonic seizures. His MRI was unremarkable except for limited small vessel disease (Figure 1).

On physical examination, he was alert and oriented to person, place, and time. His speech was fluent, not dysarthric. He had an appropriate fund of knowledge, aware of current events, and naming and repetition was normal. Memory was intact. His cranial nerve examinations were unremarkable. His sensation in the upper and lower extremities was intact bilaterally. His deep tendon reflex in the upper and lower extremities was normal and symmetric. Motor examination showed a resting tremor that was worse on the right compared to the left. He also presented with spasms of the face that appeared to be blepharospasm. Based on these findings, it was highly suspicious that the patient had Meige syndrome, which is a combination of blepharospasm and oromandibular dystonia.

For the patient’s treatment, he was taken off of chlorpromazine, an antipsychotic medication, and his symptoms drastically improved. This may support the theory that neuroleptic medications result in heightened central dopaminergic activity, leading to dystonia. However, his improvement did not last long, and his symptoms got worse. He was already on levetiracetam 250 mg daily and the dosage was increased to 500 mg twice a day to see if there were any improvements. The patient followed up after a month, and symptoms got drastically worse and he had difficulties talking. He was taken off levetiracetam and put on carbidopa/levadopa 25-100 mg three times a day to see if there are any improvements. After a month, the patient followed up with tremors of the head and body and difficulty in speaking. Carbidopa/levodopa was not effective. At that time, Meige syndrome was diagnosed and he was put on baclofen 5 mg every evening and referred to a movement disorder specialist a month later for further treatment and evaluation.

The patient responded well to the baclofen within one week. A referral to a movement disorder specialist was made. The specialist agreed with the diagnosis, and the patient has done well since the initial presentation. The patient was advised to get botox injections but denied it because baclofen alleviated his symptoms. Currently, he does not have signs of ongoing dystonia or other movement disorders and will continue to take baclofen.

## Discussion

Here is a case of a patient who presented with Meige syndrome, which is a combination of blepharospasm and oromandibular dystonia. Common symptoms are frequent eye spams and contraction that result in the inability of the eyes to open and in severe cases can lead to functional blindness. Other symptoms involve involuntary contraction of mouth and jaw jerks leading to twitches in the cheeks, lips, and tongue protrusion.

There are no special tests to diagnose Meige syndrome. Diagnosis is based upon a thorough patient history, external observations, physical examination, and clinical findings of known symptoms.

The patient first reported spasms that stemmed from his stomach ascending to his throat. His primary care provider prescribed chlorpromazine for esophageal spasms. There is a case of a woman who was on antipsychotic drugs that included chlorpromazine for two years and developed Meige syndrome [5]. Although our patient reported symptoms of spasms before being prescribed this neuroleptic medicine, chlorpromazine may have worsened his condition. He had increased muscle spasms that affected his eating and speech and improved upon stopping chlorpromazine.

In getting to the patient’s diagnosis, he was first treated with levetiracetam as seizure was a concern. That did not improve his condition and he was given carbidopa/levodopa, a medication to treat Parkinson’s disease. His condition did not improve after a month follow-up. Based on the patient history alone, it was difficult to arrive at the correct diagnosis until the patient presented to the office with symptoms. A trial of baclofen was initiated, and the patient’s condition drastically improved.

## Conclusions

Meige syndrome should always be a consideration when someone presents with symptoms suggestive of blepharospasm and oromandibular dystonia. It can occur in association with other diseases, including Parkinson's disease.
